# Standardized human granulocyte isolation using Mono-Poly™ resolving medium: protocol development, validation, and comparison with Lymphoprep™

**DOI:** 10.1016/j.mex.2026.104213

**Published:** 2026-10-16

**Authors:** Othman Eldalal, Steven Lockhart, Huan Liu, John-Paul R. Cooper, Dinesh Babu, Arno G. Siraki

**Affiliations:** a Faculty of Pharmacy and Pharmaceutical Sciences, College of Health Sciences, University of Alberta, Edmonton, Canada; b Department of Drug Technology, Faculty of Medical Technology, Derna, Libya; c Li Ka Shing Institute of Virology, Department of Medical Microbiology and Immunology, University of Alberta, Edmonton, Canada

## Abstract

High purity neutrophil preparations are required for studying neutrophil biology and function. Although density gradient centrifugation is the most commonly used isolation technique, the choice of gradient medium can significantly affect cell purity and downstream applications. The stepwise method of using Mono-Poly™ Resolving Medium (M-PRM) for human peripheral blood granulocyte isolation is presented here alongside the Lymphoprep™ protocol, as these are the two commercially available density-gradient media selected for comparison in this study. Both protocols were optimized and validated by flow cytometric analysis using sequential FSC/SSC gating, singlet discrimination, and CD15-positive granulocyte phenotype identification. The use of M-PRM resulted in significantly higher average granulocyte purity (95.3%) than Lymphoprep™ (73.9%). This comparative protocol provides a practical guide for selecting an appropriate density gradient medium based on experimental requirements and offers a standardized workflow for obtaining granulocyte-enriched cell preparations suitable for downstream immunological and functional studies.

## Graphical abstract

**Figure:**
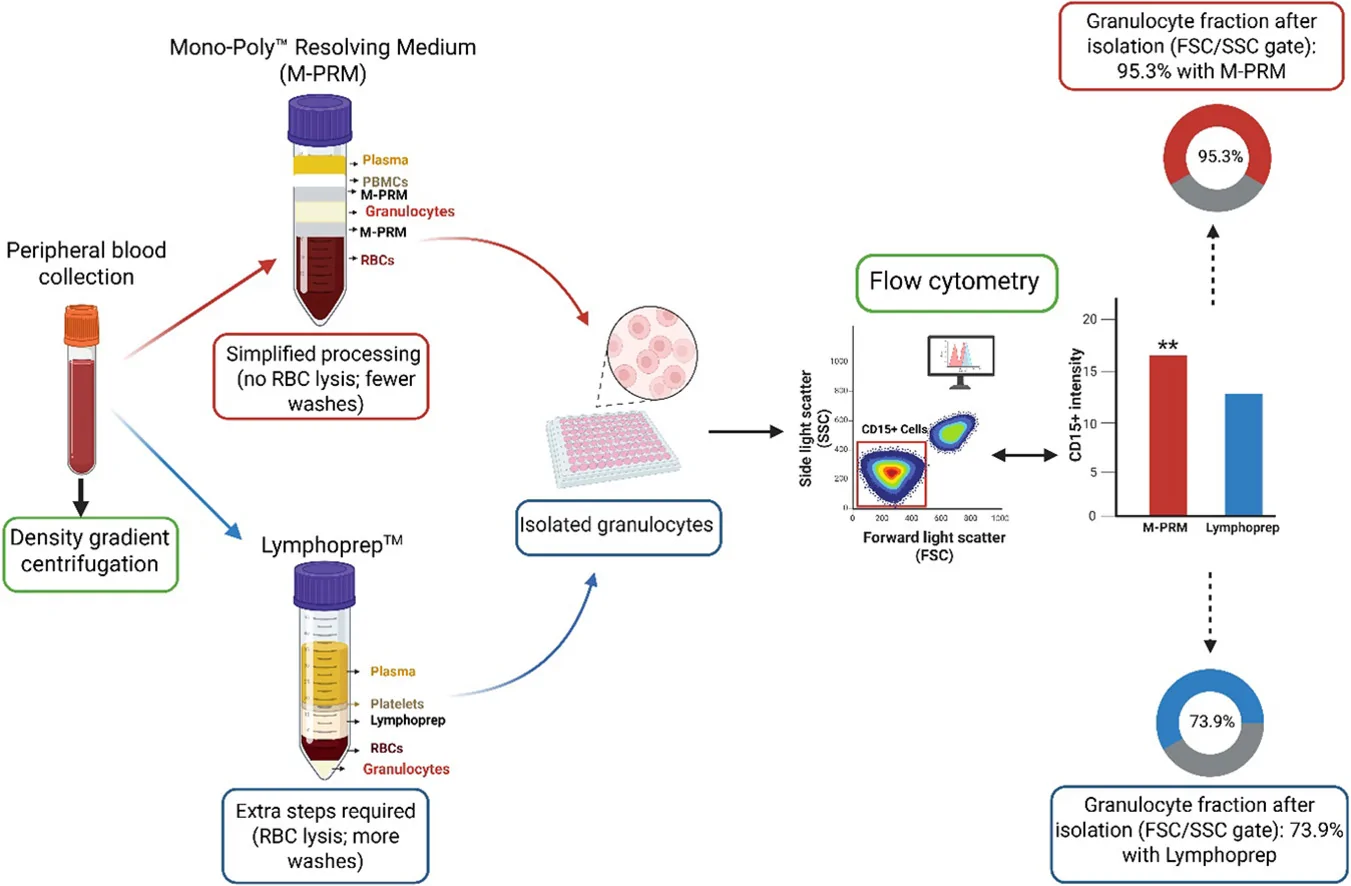

**Specifications Table**

**Table:** 

**Subject area**	Pharmacology, Toxicology and Pharmaceutical Sciences
**More specific subject area**	Immunology; Cell Biology;
**Name of your method**	Standardized protocol for human neutrophil isolation using Mono-Poly™ Resolving Medium (M-PRM), with comparative evaluation against Lymphoprep™
**Name and reference of original methods**	Method 1: Lymphoprep™ density gradient neutrophil isolation protocol (STEMCELL Technologies). Method 2: Mono-Poly™ Resolving Medium (M-PRM) density gradient protocol (MP Biomedicals). The M-PRM protocol was standardized and optimized, with direct comparison with the established Lymphoprep™ method using flow cytometric assessment of neutrophil purity.
**Resource availability**	Materials, reagents, and equipment are listed in Table 1.
**Method validation**	Validation of the standardized M-PRM protocol by comparative flow cytometric analysis of neutrophil purity using FSC/SSC gating, singlet discrimination, and a CD15-positive granulocyte phenotype identification, with direct comparison to the established Lymphoprep™ method.

## Background

Neutrophils are the most abundant circulating leukocytes, which represent approximately 40–75% of whole white blood cells and constitute >90% of circulating granulocytes in healthy human blood compared to eosinophils and basophils [1]. Neutrophils are first responders and serve as the first line of defense in innate immune system against invading pathogens [2]. They mediate host defense through several mechanisms such as phagocytosis, production of reactive oxygen species (ROS), degranulation, cytokine secretion, and the formation of neutrophil extracellular traps (NETs) [3]. Therefore, isolating granulocyte populations and accurately characterizing their cellular composition are important for studies of neutrophil biology and function. Conducting in vitro studies of neutrophils is still challenging, because of their short lifespan, inability to proliferate in culture, and high sensitivity to environmental and mechanical stimuli that can cause unintended activation and alter their phenotype [3]. Freshly isolated human neutrophils are still the most physiologically relevant model for studying native neutrophil biology. For in vitro studies, neutrophils should be isolated as soon as possible (preferably within 1 hour) after peripheral blood collection, to keep their physiological state and reduce activation and apoptosis [4,5].

**Table 1:** Materials, reagents, and equipment used in this study.

Reagent/Material	Source	Catalog Number	Notes
RPMI-1640 Medium	Thermo Fisher Scientific (Ottawa, Canada)	12633012	Cell culture medium
Phosphate-Buffered Saline (PBS)	Thermo Fisher Scientific (Ottawa, Canada)	AM9624	Washing buffer
Fetal Bovine Serum (FBS)	Thermo Fisher Scientific (Ottawa, Canada)	A5256701	Cell culture supplement
Lymphoprep™ Density Gradient Medium	STEMCELL Technologies (Vancouver, Canada)	18061	Density gradient medium for neutrophil isolation
Mono-Poly™ Resolving Medium (M-PRM)	MP Biomedicals (Irvine, USA)	091698049	Density gradient medium for neutrophil isolation
Red Blood Cell (RBC) Lysis Buffer	Prepared in-house	N/A	10× stock: 1.55 M NH₄Cl, 120 mM NaHCO₃, 1 mM EDTA
Paraformaldehyde	Thermo Fisher Scientific (Ottawa, Canada)	AAJ19943K2	Prepared as 4% fixative for flow cytometry
PE-Conjugated Mouse Anti-Human CD15 Antibody	BD Biosciences (CA, USA)	555402	Flow cytometry staining of granulocytes
BD Vacutainer® K2 EDTA Blood Collection Tubes	BD Biosciences (CA, USA)	366664	Peripheral blood collection
Sterile 96-Well Round-Bottom Tissue Culture Plates	Corning Costar (Thermo Fisher Scientific; Ottawa, Canada)	3799	Cell staining and fixation
15 mL Sterile Conical Tubes	Corning/Falcon or equivalent (NY, USA)	N/A	Density gradient separation
50 mL Sterile Conical Tubes	Corning/Falcon or equivalent (NY, USA)	N/A	Density gradient separation and washing
Flow staining buffer (PBS + 2% FBS)	Prepared in-house	N/A	Antibody dilution and washing
Complete RPMI-1640 Medium Trypan blue stain 0.4% LSRFortessa™ Flow Cytometer Allegra X-22R refrigerated centrifuge	Prepared in-house Thermo Fisher Scientific (Ottawa, Canada) Becton Dickinson. (NJ, USA) Beckman Coulter, Brea, CA, USA.	N/A 15250061 N/A 392187	RPMI-1640 supplemented with 10% FBS (and antibiotics) cell viability staining Flow cytometric analysis of granulocytes purity. All centrifugation steps

Among the available neutrophil isolation methods, density gradient centrifugation is still the most used approach since it is simple, cost-effective, scalable, and preserves cell viability [6]. Other approaches, including red blood cell (RBC) sedimentation, immunomagnetic separation, fluorescence-activated cell sorting (FACS), and microfluidic technologies, have also been developed, but these approaches commonly are more technically demanding, need specialized equipment or may enhance the risk of unwanted neutrophil activation, making them unsuitable for routine use in many laboratories [[7], [8], [9], [10], [11]].

To support our choice of density gradient centrifugation method for protocol development, we performed a literature survey of primary research articles indexed in PubMed and Scopus between 2000 and 2025. As shown in Fig. 1, density gradient centrifugation was the most frequently used neutrophil isolation method across all intervals, supporting its continued role as the preferred approach for routine laboratory applications.

**Fig. 1:**
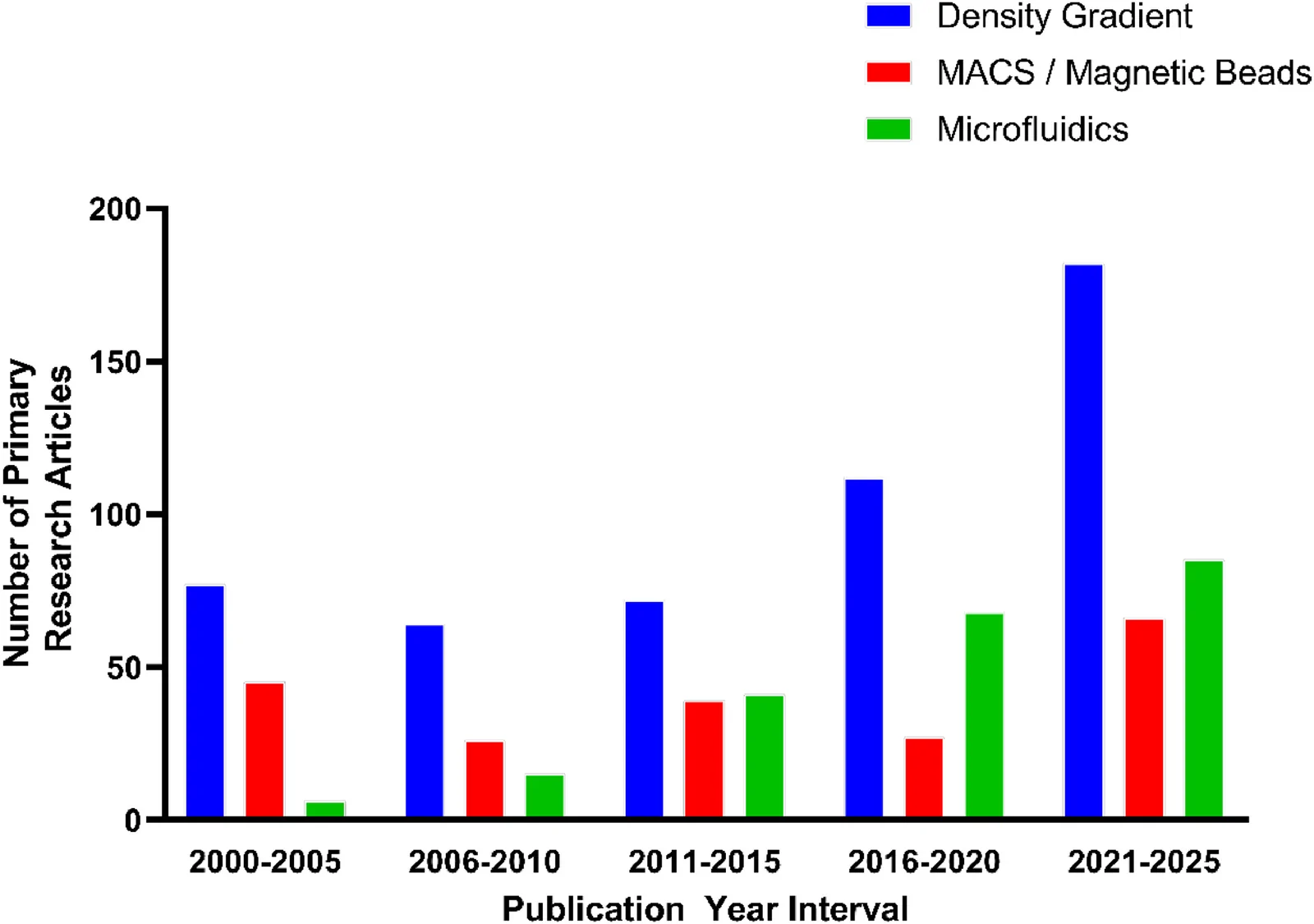
**Frequency of primary research articles describing neutrophil isolation by methodological category, between 2000–2025.** The records were retrieved from PubMed and Scopus on April 15, 2026, using category-specific Boolean queries (full strings provided in Supplementary Table S for density gradient centrifugation (blue), magnetic bead–based selection (red), and microfluidic (green) platforms. The documents tagged as reviews were excluded using a search query; titles and document types were manually screened, and individual records were manually verified for relevance. The counts within each category were de-duplicated across the two databases by DOI/title matching and grouped in publication-year intervals.

Several commercially available density gradient media are currently used for granulocyte isolation especially neutrophil, including Ficoll-Paque™, Lymphoprep™, Mono-Poly™ Resolving Medium (M-PRM) and Percoll™ [[12], [13], [14]]. These media differ in density, separation principle, workflow, and cell recovery, all of which may affect granulocyte purity and the quality of isolated cell preparations [[12], [13], [14], [15]]. This study focused on M-PRM and Lymphoprep™ because they represent two commercially available neutrophil isolation media and require similar laboratory infrastructure while employing different density-gradient strategies.

Although Lymphoprep™ is a well-established and extensively characterized isolation medium [16,17], M-PRM has received a limited methodological evaluation and protocol standardization despite its reported use for neutrophil isolation. To address this methodological gap, we developed an optimized protocol for granulocyte isolation using M-PRM and compared its performance with the commonly used Lymphoprep™ method using flow cytometric assessment of granulocyte purity. In addition to the optimized M-PRM workflow, this article also provides detailed protocols for Lymphoprep™ isolation and a standardized strategy for granulocyte purity assessment to facilitate reproducible implementation in other laboratories.

## Method details

### Before you begin

**Important:** Granulocytes especially neutrophils are highly sensitive to environmental and mechanical stimuli that may cause unintended activation, change surface marker expression, and compromise downstream functional assays [5]. To maintain cell viability and keep the cells in resting state, the following precautions should be observed throughout the isolation procedure:

• Due to the short lifespan of neutrophils, all experimental procedures must be begun immediately after collection of blood samples to maintain cell viability and functional integrity.
• Peripheral blood was collected into sterile BD Vacutainer® K2 EDTA blood collection tube to avoid coagulation and protect leukocyte integrity during sample handling. The samples were handled within the recommended time frame to maintain cell viability.
• Neutrophils are highly sensitive to environmental conditions and can be activated by mechanical stress or chemical contaminants. To reduce unwanted activation during isolation, all reagents, buffers, media, and labware must be pyrogen-free. Additionally, aerosol-resistant barrier pipette tips and sterile, pyrogen-free pipettes were used throughout the procedures.
• All cell handling procedures were conducted within a biosafety cabinet equipped with HEPA-filtered airflow to minimize contamination and protect against aerosol-induced activation. Standard biosafety practices were followed, including the use of personal protective equipment (gloves, lab coats, and safety glasses for eye protection) and aerosol control devices (biosafety cabinet).
• To prevent unintended neutrophil activation, unnecessary shaking or vortexing of cells was strictly avoided, as mechanical stress can induce priming, promote excessive activation, and lead to the formation of cell clumps compromising experimental results.
• Blood samples and isolated cells should be maintained at room temperature (RT) and physiological pH throughout the procedure. All isolation solutions were prepared without divalent cations (Ca²⁺ and Mg²⁺) to avoid cellular activation.

### Human participants

#### Ethics statement

The participants were recruited as volunteers for this study. This neutrophil isolation study was approved by the Human Research Ethics Board (HREB) at the University of Alberta (Pro00008304), and informed written consent was obtained from all the participants. The peripheral blood samples were collected from six healthy individuals with no known co-morbidities.

### Isolation of granulocytes using Mono-Poly™ resolving medium (M-PRM)

#### Separation principle

M-PRM is a density gradient medium composed of a polysaccharide (Ficoll 400) and a radiopaque contrast agent (Hypaque). It has an optimized density (1.114 ± 0.002 g/mL) facilitating the separation of mononuclear and polymorphonuclear leukocytes into distinct bands [18]. Undiluted, anticoagulant-treated blood is carefully layered over M-PRM before centrifugation [18]. Following centrifugation, peripheral blood mononuclear cells (PBMCs), including monocytes and lymphocyte populations, form an upper band at the plasma/M-PRM interface, whereas the granulocyte fraction, predominantly comprising neutrophils in healthy peripheral blood, forms a second band below. (Fig. 2). Erythrocytes, being denser will pass through the gradient to form a pellet at the bottom [18]. This differential migration results in two clearly defined leukocyte bands that are relatively free of erythrocytes, enabling the efficient collection of granulocytes with minimal contamination with RBCs [19].

**Fig. 2:**
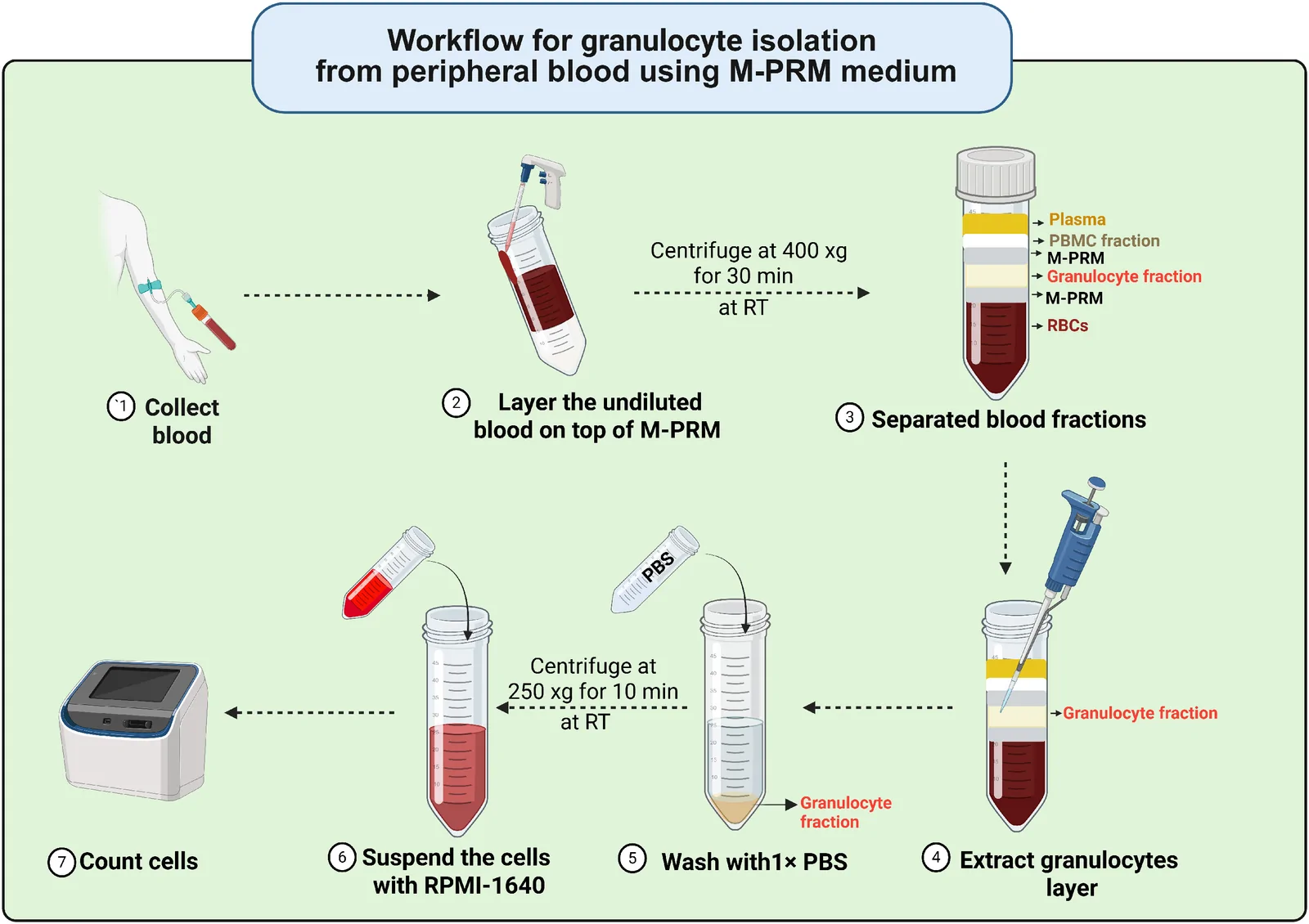
**Isolation of granulocytes from whole blood using M-PRM density separation medium**. Two fractions are visible after centrifugation, where Fraction 1 contains the PBMC fraction, and Fraction 2 consists of granulocytes. The erythrocyte pellet is at the bottom of the tube.

#### Protocol

1. Blood was collected into BD Vacutainer® K2 EDTA blood collection tube from healthy donors.
2. Before use, the M-PRM solution was inverted 2–3 times to mix well.

**Note**: Do not vortex the M-PRM solution, as excessive agitation may introduce bubbles and disrupt density gradient formation.

3. 7 mL of M-PRM was placed in a 15 mL centrifugation tube.
4. 7 mL of undiluted blood was carefully layered on top of the M-PRM.

**Critical step**: The blood should be slowly layered along the wall of the tube to avoid mixing with the separation medium, which may reduce separation efficiency and granulocyte purity.

5. The tubes were centrifuged at 400 × g. with acceleration (Acc) set to 7, deceleration (Dec) set to 0, and Brake set to 0 for 30 min at RT.

**Critical step**: The centrifuge brake should remain off to preserve the density gradient and prevent disruption of the granulocyte layer.

6. The plasma layer is discarded carefully without disturbing the granulocyte layer.
7. The granulocyte layer was carefully collected with a pipette and transferred into another centrifugation tube.

**Note**: Aspirate only the visible granulocyte layer. Avoid collecting excess M-PRM or nearby cell layers to reduce contamination.

8. 1× PBS was added to the isolated cells to wash away residual M-PRM.
9. The cells were centrifuged at 250 × g (Acc: 7, Dec: 0, Brake: 0) for 10 min at RT, and the supernatant was discarded.

**Note**: A single wash is generally sufficient to remove residual M-PRM. Additional washing may reduce cell recovery and should be performed only if necessary.

10. The pellet was resuspended in 5 mL pre-warmed complete RPMI-1640 media or 1× PBS.
11. The cells were counted using trypan blue.

**Safety note:** Trypan blue is a hazardous dye and should be handled and disposed of according to safety guidelines.

12. The cell counting was performed within minutes to reduce trypan blue-induced cytotoxicity.

**Note**: Prolonged exposure to trypan blue may reduce cell viability and affect accurate viability measurements.

### Isolation of granulocytes using the Lymphoprep™ medium

#### Separation principle

Lymphoprep™ is a medium composed of 9.1% (w/v) sodium diatrizoate, and 5.7% (w/v) polysaccharide balanced with water, with a specific density of 1.077 g/ mL. Its relatively high density facilitates the aggregation and sedimentation of erythrocytes during centrifugation [17,20]. It is denser than lymphocytes, monocytes, and platelets but less dense than granulocytes and erythrocytes. Upon centrifugation, erythrocytes and most granulocytes sediment to form a pellet at the bottom of the tube, while plasma and platelets remain in the top layer (Fig. 3). Peripheral blood mononuclear cells (PBMCs) accumulate at the interface between the plasma and the density gradient medium, where the density of the cells matches that of the surrounding medium [20,21].

**Fig. 3:**
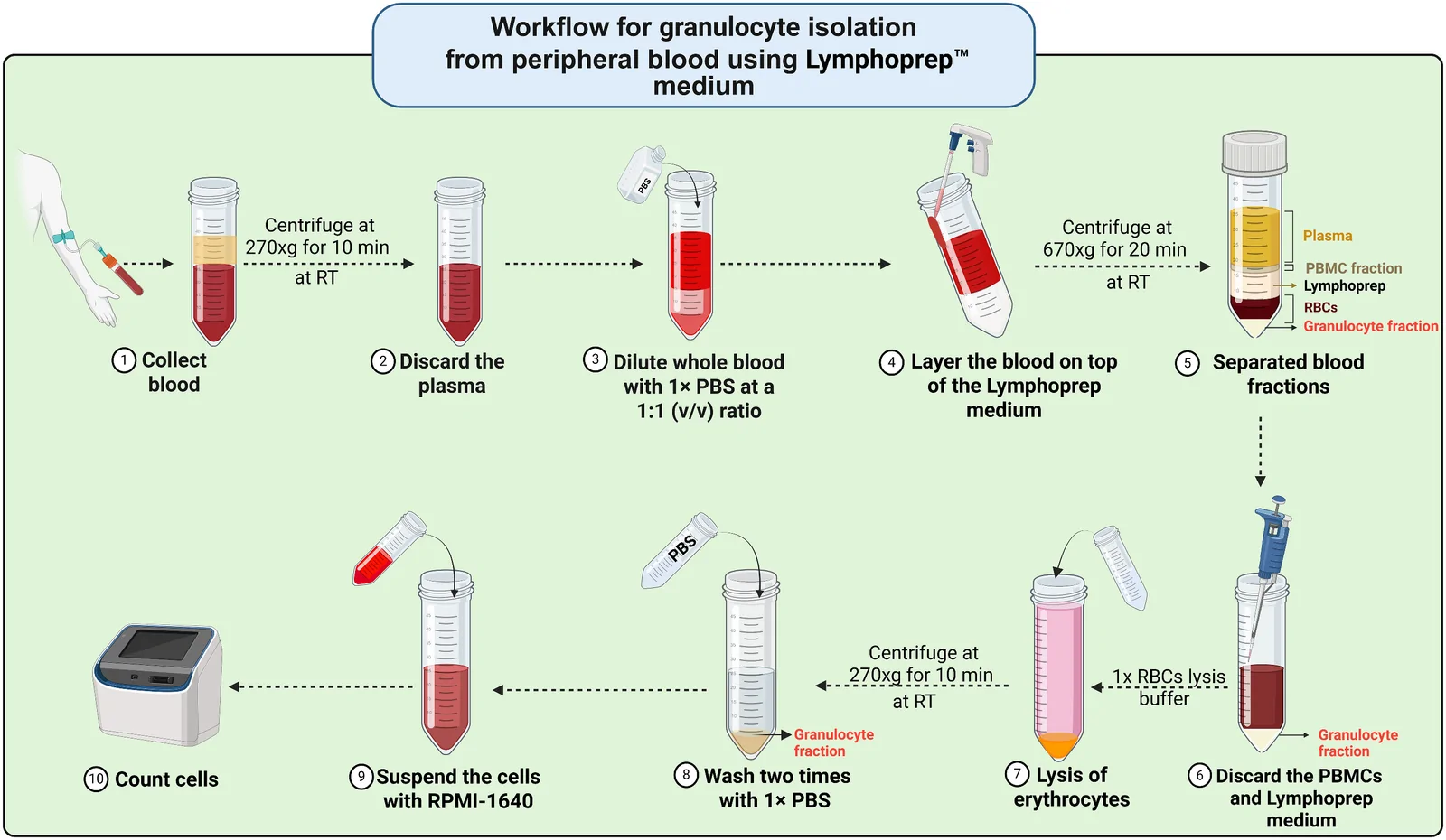
**Isolation of granulocytes from whole blood using Lymphoprep™ density separation medium**. The white coat containing the desired granulocytes is located at the bottom attached to RBCs, separated from the medium, plasma, and PBMC fraction.

#### Protocol

1. Blood was collected into K2 EDTA anticoagulant-coated tubes from healthy donors.
2. The blood cells were centrifuged at 270 ×g (Acc: 10, Dec: 0, and Brake: 0) 10 min at RT.
3. The plasma layer was carefully discarded or decanted (for possible future use, Fig. 3).
4. The blood sample was diluted with 1× PBS to reduce viscosity (15 mL blood + 15 mL PBS is a 1:1 (v/v) dilution).
5. 15 mL of Lymphoprep™ medium was added to a 50 mL centrifugation tube.
6. The diluted blood (up to 30 mL) was loaded on top of Lymphoprep™ by gently running it down the side of the centrifugation tube.

**Note**: Care should be taken to avoid mixing the blood with the density gradient medium, since disruption of the interface may reduce granulocyte recovery and purity.

7. The samples were centrifuged at 670 × g (Acc: 10, Dec: 0, Brake: 0) for 20 min at RT.

**Critical step**: The centrifuge brake should remain off during density gradient centrifugation to maintain the gradient interface and ensure efficient cell separation.

8. The granulocytes were allowed to settle at the bottom of the tube.
9. The PBMC layer and the Lymphoprep™ were carefully aspirated from the top of the tube.

**Note**: Care should be taken not to disturb the granulocyte pellet during aspiration, as this may reduce cell recovery.

10. 1× RBC lysis buffer was added to bring the total volume to 50 mL.
11. The mixture was allowed to sit for 10 min at RT until the solution became darkened, indicating RBC lysis.

**Note**: The RBC lysis step should not be extended beyond the recommended incubation time, since prolonged exposure may reduce granulocyte viability and change cell phenotype.

12. The cells were centrifuged at 270 × g (Acc: 10, Dec: 0, Brake:0) for 10 min at RT.
13. The lysed RBCs were carefully discarded.
14. 1× PBS was slowly added down the side of the centrifugation tube. The centrifugation tube was gently agitated to rinse the pellet, and the supernatant was carefully aspirated without a secondary centrifugation step.
15. The washing step was repeated one more time until all the debris and residual RBCs were removed.

**Note**: Handle the cells gently during the washing steps, since harsh pipetting or repeated resuspension could lead to granulocyte activation and affect overall cell quality.

16. The granulocytes were resuspended in 5 mL of pre-warmed complete RPMI-1640 medium.
17. The granulocytes were counted using the trypan blue.
18. The cell counting was achieved quickly since trypan blue can kill cells over time.

## Method validation

### Flow cytometry analysis of isolated granulocytes

Flow cytometry was used to assess the granulocyte fraction of the isolated cell preparations based on light-scatter characteristics and cell-surface marker expression [22]. Neutrophils constitute the major circulating granulocyte population and are therefore expected to represent the predominant cell type within the isolated granulocyte fraction [1]. CD15, also known as the Lewis X antigen, was used as a granulocyte-associated surface marker. However, because both neutrophils and eosinophils can express CD15, CD15 positivity was interpreted as confirming a CD15-positive granulocyte phenotype [23,24].  

Antibody Staining and Fixation of Isolated Cells for Flow Cytometry Analysis:

1. 1 × 10^6^ cells suspended in RPMI-1640 medium supplemented with 10% fetal bovine serum (FBS) and 1% penicillin-streptomycin were seeded in a sterile 96-well round-bottom plate.
2. The plate was centrifuged at 430 × g (Acc: 10, Dec: 0, Brake: 0) for 3 min at RT.
3. The media was carefully discarded.

**Note**: Avoid disturbing the cell pellet when discarding the supernatant, as granulocytes form a relatively loose pellet.

4. Flow staining buffer (PBS + 2% FBS) was prepared in house for antibody staining.
5. PE-conjugated mouse anti-human CD15 antibody was diluted at 1:25 (1 µL of antibody + 24 µL of flow staining buffer) in an Eppendorf tube.

**Note**: Prepare antibody dilutions immediately before use and protect fluorophore-conjugated antibody dilutions from light.

6. 25 µL of the diluted antibody was added to each well.
7. The cells were gently mixed with the antibody using a multichannel pipette to minimize bubble formation.

**Critical step**: Gentle mixing ensures uniform antibody staining and minimizes mechanical activation or cell loss.

8. The plate was covered to protect from light and incubated for 30 min at 4 °C.

**Note**: Incubate for 30 min at 4 °C to preserve surface antigen expression and minimize receptor modulation and internalization.

9. Flow staining buffer (175 µL) was added to each well to bring the volume to 200 µL.
10. The plate was centrifuged at 430 × g (Acc: 10, Dec: 10, Brake: 0) for 3 min at RT.
11. The supernatant was carefully discarded.
12. 200 µL of 4% paraformaldehyde was added to the cells and mixed well in the 96-well plate and incubated for 30 min at 4 °C, protected from light, to fix the cells.

**Critical step:** Store 4% paraformaldehyde in sealed, single use aliquots and use a fresh aliquot for each experiment. Minimize exposure to air and avoid repeated freeze-thaw cycles. Discard any precipitated or cloudy solution to maintain consistent fixation.

13. The cells were again centrifuged at 430 × g (Acc: 10, Dec: 10, Brake: 0) for 3 min at RT.
14. Discard the supernatant (which contains paraformaldehyde).

**Safety note**: Paraformaldehyde is hazardous and should be handled and disposed of according to safety guidelines.

15. Resuspend the cells with 200 µL of flow staining buffer and mix gently.
16. The cells were transferred into flow cytometry tubes and were kept protected from light.
17. Finally, the samples were either subjected to flow cytometry analysis or stored in the refrigerator to be analyzed at a later time (maximum 1-day delay) (Fig. 4).

**Fig. 4:**
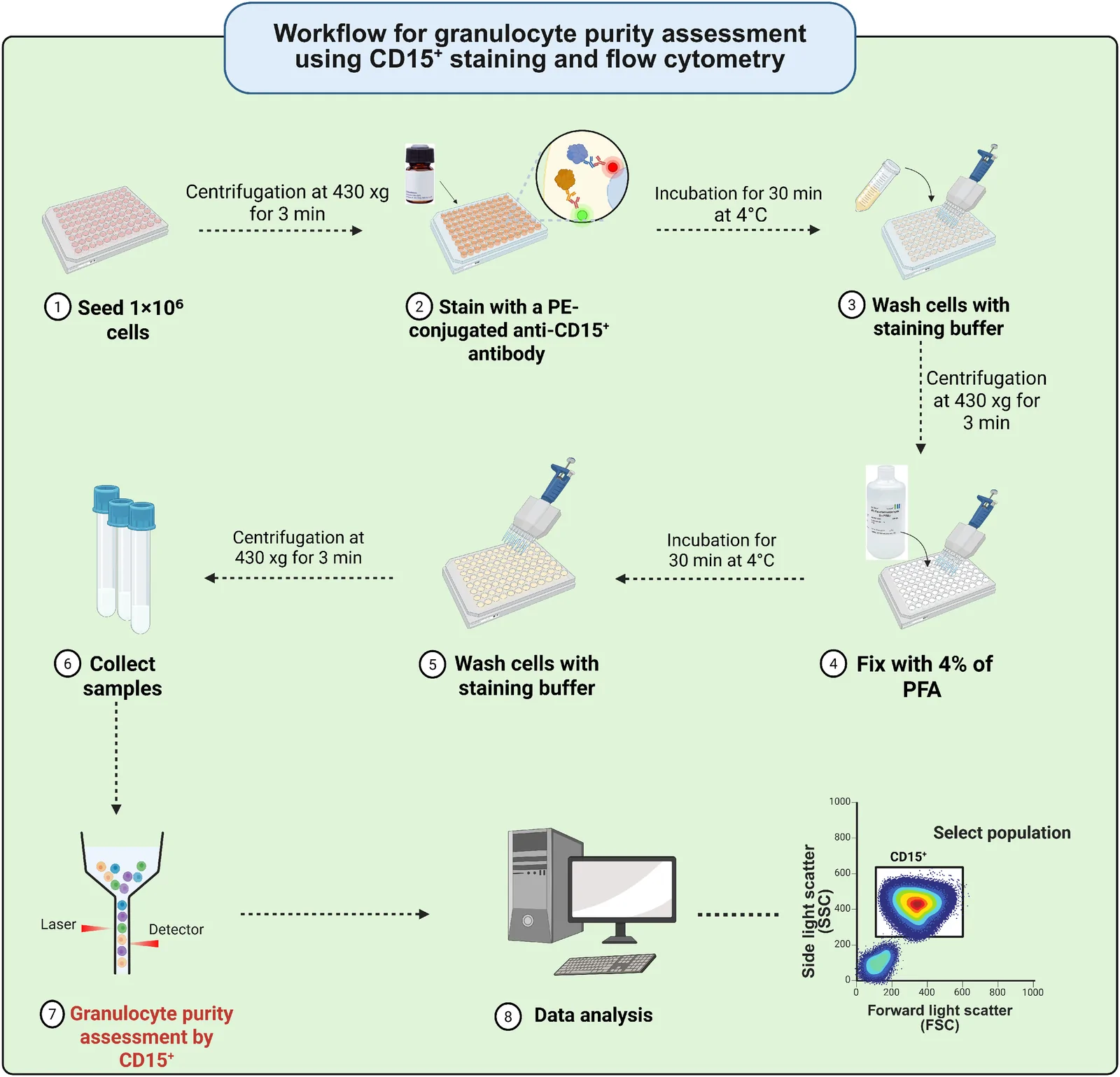
**Workflow for flow cytometric analysis of granulocytes:** A total of 1 × 10⁶ cells were stained with PE-conjugated CD15^+^ antibody, fixed with 4% paraformaldehyde and analysed using a flow cytometer.

## Validation result

Flow cytometry was used to compare the cellular composition of fractions isolated using M-PRM and Lymphoprep™. Events with granulocyte-associated forward- and side-scatter characteristics were first identified using FSC-A versus SSC-A. Singlets were then selected using FSC-H versus FSC-A to exclude doublets and aggregates, followed by assessment of CD15 expression within the singlet granulocyte gate. Representative flow cytometry gating strategies and population frequencies for both isolation methods are presented in Fig. 5. Initial primary gating was based on (FSC-A/SSC-A) to gate total granulocyte population while excluding small debris, yielding 95.6% granulocytes for M-PRM compared with 74.1% for Lymphoprep™ (Fig. 5A1, B1). Singlet discriminationFSC-H vs FSC-A) was used to gate single cells and exclude cell aggregates, confirming high single-cell frequencies of 100.0% and 99.8% within these primary gates for M-PRM and Lymphoprep™, respectively (Fig. 5A2, B2). Phenotypic characterization using SSC-A vs CD15-associated fluorescence intensity was used to gate granulocytes while excluding contaminating cells such as RBCs or PBMCs, showing that 99.9% of gated cells were across both preparations (Fig. 5A3, B3). Across three independent experiments, the proportion of events within the granulocyte gate was significantly higher following isolation with M-PRM than with Lymphoprep™ (95.3% ± 0.25% versus 73.9% ± 0.62%, respectively; *P* < 0.001; Fig. 5C). These findings demonstrate that M-PRM produced a more highly enriched granulocyte fraction than Lymphoprep™.

**Fig. 5:**
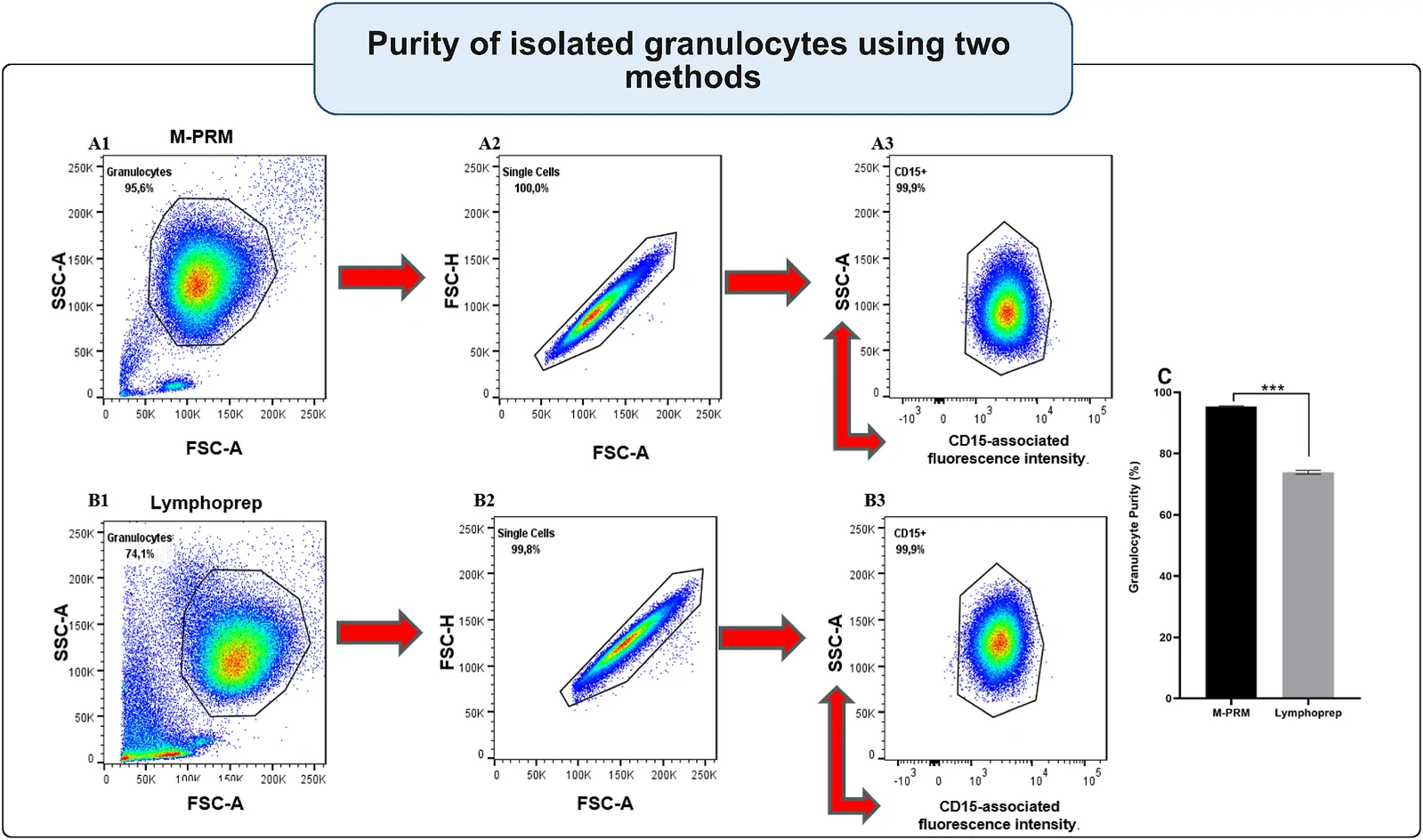
Flow cytometric markers and gating strategies were used to analyze granulocyte populations following isolation using M-PRM and Lymphoprep™. (A1-A3) Representative flow cytometry gating strategies for M-PRM isolation. (B1-B3) Representative gating sequence for Lymphoprep™ isolation. Isolated cells were stained with anti-CD15 following standard protocols. Total granulocytes (A1, B1) were gated based on FSC-A and SSC-A, producing initial purities of 95.6% and 74.1% for M-PRM and Lymphoprep™, respectively. Singlet cells (A2, B2) were gated from the granulocyte population using FSC-H and FSC-A to exclude cell aggregates. Target granulocyte identity was confirmed by gating SSC-A and CD15^+^ cells within the singlet population (A3, B3), showing 99.9% CD15^+^ positivity across both preparations. (C) Granulocyte purity was significantly higher following isolation using M-PRM than Lymphoprep™. Data are presented as mean ± SD from three independent experiments (n = 3). Statistical analysis was performed using an unpaired *t*-test; ***P < 0.001.

## Practical comparison of the two protocols

Both M-PRM and Lymphoprep™ successfully isolated granulocytes from human peripheral blood; however, the two protocols differed in workflow and initial granulocyte purity. M-PRM produced significantly higher initial granulocyte purity of approximately 95.3% than Lymphoprep™ (73.9%) without the need for blood dilution and RBC lysis which simplifies the isolation procedure. The absence of an RBC lysis step is a practical advantage of the M-PRM protocol since previous studies have shown that RBC lysis procedures affect neutrophil activation status, surface marker expression, and downstream functional responses [25]. Consequently, avoiding RBC lysis may help preserve the native neutrophil phenotype and reduce sample handling. Conversely, the Lymphoprep™ protocol requires blood dilution, RBC lysis, and additional washing steps to isolate granulocytes. Both M-PRM and Lymphoprep™ permit recovery of a PBMC-containing fraction in addition to the granulocyte fraction. Therefore, simultaneous PBMC recovery is not unique to Lymphoprep™. The principal practical distinction observed in the present comparison is that M-PRM produced a substantially more enriched granulocyte fraction while requiring fewer processing steps and avoiding RBC lysis. The potential impact of RBC lysis on neutrophil activation and function warrants further investigation. The main practical differences between the two protocols are summarized in Table 2.

**Table 2:** Summary and comparison of M-PRM and Lymphoprep™ for granulocyte isolation.

Feature	Mono-Poly™ Resolving Medium (M-PRM)	Lymphoprep™
Whole blood preparation	Whole blood used directly	Dilution with PBS required
Density gradient medium	M-PRM (1.114 ± 0.002 g/mL)	Lymphoprep™ (1.077 g/mL)
Granulocyte recovery	Recovered as a distinct granulocyte layer	Recovered with erythrocytes
Red blood cell (RBC) lysis	Not required	Required
Average granulocyte purity	95.3%	73.9%
PBMC recovery	PBMCs recovered as a separate upper layer	PBMCs recovered as a separate upper layer
Processing workflow	Fewer processing steps	More processing steps
Relative cost	Lower	Higher
Recommended application	Studies prioritizing high initial granulocyte purity and PBMCs	Studies requiring both granulocytes and PBMCs

## Study limitations

Although there are clear differences in granulocyte purity between the M-PRM and Lymphoprep™ methods, this study has several limitations. First, granulocytes were initially identified based on their FSC/SSC characteristics. CD15 was subsequently used as a granulocyte-associated surface marker. Because CD15 is expressed by both neutrophils and eosinophils, CD15 positivity alone was not interpreted as establishing neutrophil-specific identity or purity. Furthermore, we assessed CD15 positivity within the gated granulocyte population but did not evaluate neutrophil function. Although higher initial purity and reduced handling with the M-PRM method may suggest better preservation of resting physiology, functional assays, such as ROS production, phagocytosis, degranulation, and NET formation, are needed to confirm whether these differences affect effector functions. Second, neutrophil activation status was inferred from the literature on RBC lysis and handling stress rather than directly measured. Including activation markers, such as CD11b upregulation, CD62L shedding, and increased CD66b expression, would provide stronger evidence for the maintenance of a resting phenotype [25]. Finally, the samples used in this study were from healthy male donors only. Neutrophils from patients with inflammatory or hematological conditions may respond differently to isolation, limiting generalizability to disease settings. Future studies involving functional profiling, direct activation markers, and patient-derived samples would strengthen these findings and better guide the method selection for specific applications.

## Conclusion

M-PRM produced a significantly more enriched initial granulocyte preparation than Lymphoprep™ under the conditions tested. Additional enrichment and validation would be needed if a highly purified neutrophil preparation is required following Lymphoprep™ isolation. The detailed protocols and validation workflow presented here provide a reproducible approach for human neutrophil isolation and purity assessment. By highlighting the practical differences between the two density gradient media, this work may help researchers in selecting the most appropriate isolation method for their experimental objectives.

## Credit author statement

**Othman Eldalal**: Conceptualization, Methodology, Validation, Formal analysis, Investigation, Writing – Original Draft, Writing – Review & Editing, Visualization, Project administration. **Steven Lockhart**: Methodology, Investigation, Validation. **Huan Liu**: Methodology, Investigation, Validation. **John-Paul R Cooper**: Data curation, Visualization. **Dinesh Babu**: Conceptualization, Validation, Writing – Review & Editing. **Arno G. Siraki**: Conceptualization, Resources, Writing – Review & Editing, Funding acquisition.

## Declaration of competing interests

The authors declare that they have no known competing financial interests or personal relationships that could have appeared to influence the work reported in this paper.

## Data Availability

Data will be made available on request.

## References

[1] Kostenis E., Ulven T. (2006). Emerging roles of DP and CRTH2 in allergic inflammation. Trends. Mol. Med..

[2] Hirz T., Dumontet C. (2016 Mar 25). Neutrophil isolation and analysis to determine their role in lymphoma cell sensitivity to therapeutic agents. J. Vis. Exp..

[3] Blanter M., Gouwy M., Struyf S. (2021). Studying neutrophil function in vitro: cell models and environmental factors. J. Inflamm. Res..

[4] Siemsen D.W., Malachowa N., Schepetkin I.A., Whitney A.R., Kirpotina L.N., Lei B., et al. Chapter 3 neutrophil isolation from nonhuman species. Methods in Molecular biology. 1124. doi:10.1007/978-1-62703-845-4_3. 10.1007/978-1-62703-845-4_3 PMC6777848 24504944

[5] Blanter M., Cambier S., De Bondt M., Vanbrabant L., Pörtner N., Abouelasrar Salama S. (2022 Feb 10). Method matters: effect of purification technology on neutrophil phenotype and function. Front. Immunol..

[6] Alexandria G., Valerio H.P., Massafera M.P., Reis L.R., Coelho F.R., Di Mascio P. (2025). The miniaturized isolation of neutrophil granules (MING) method allowed a deep proteome mapping of human neutrophil granules Graphical Abstract. J. Leukoc. Biol..

[7] Kotz K.T., Xiao W., Miller-Graziano C., Qian W.J., Russom A., Warner E.A. (2010). Clinical microfluidics for neutrophil genomics and proteomics. Nat. Med..

[8] Warner E.A., Kotz K.T., Ungaro R.F., Abouhamze A.S., Lopez M.C., Cuenca A.G. (2011). Microfluidics-based capture of human neutrophils for expression analysis in blood and bronchoalveolar lavage. Lab. Investig..

[9] Dorward D.A., Lucas C.D., Alessandri A.L., Marwick J.A., Rossi F., Dransfield I. (2013). Technical advance: autofluorescence-based sorting: rapid and nonperturbing isolation of ultrapure neutrophils to determine cytokine production. J. Leukoc. Biol..

[10] Lee S., Kim H., Yang S. (2023). Microfluidic label-free hydrodynamic separation of blood cells: recent developments and future perspectives. Adv. Mater. Technol..

[11] Swierczak A., Pollard J.W. (2020 Jan 1). FACS isolation and analysis of human circulating and tumor neutrophils. Methods Enzym..

[12] Cui C., Schoenfelt K.Q., Becker K.M., Becker L. (2021). Isolation of polymorphonuclear neutrophils and monocytes from a single sample of human peripheral blood. Star Protoc..

[13] Sounbuli K., Alekseeva L.A., Markov O.V., Mironova N.L. (2023 Dec 1). A comparative study of different protocols for isolation of murine neutrophils from bone marrow and spleen. Int. J. Mol. Sci..

[14] Kuang Y., Parthasarathy U., Martinelli R. (2023). Protocol for density gradient neutrophil isolation and flow cytometry-based characterization from human peripheral blood. Star. Protoc..

[15] Kuhns D.B., Priel D.A.L., Chu J., Zarember K.A. (2015). 26528633.

[16] Chen H., Schürch C.M., Noble K., Kim K., Krutzik P.O., O’Donnell E. (2020). Functional comparison of PBMCs isolated by Cell Preparation tubes (CPT) vs. Lymphoprep tubes. BMC Immunol..

[17] Yeo C., Saunders N., Locca D., Flett A., Preston M., Brookman P. (2009). Ficoll-paque™ versus lymphoprep™: a comparative study of two density gradient media for therapeutic bone marrow mononuclear cell preparations. Regen. Med..

[18] Isolation of mononuclear and polymorphonuclear leukocytes from blood with density separation Media [Internet] [cited 2024 Dec 8]. Available from: https://cellculturedish.com/isolation-of-mononuclear-and-polymorphonuclear-leukocytes-from-blood-with-density-separation-media/.

[19] Lockhart S., Tabana Y., Tabatabaei Dakhili S.A., Babu D., Tran N.H., Eldalal O. (2025). A chemoproteomic strategy for identifying protein covalent binding targets of clozapine: an approach for advancing clozapine toxicity research. Toxicol. Lett..

[20] Riedhammer C., Halbritter D., Weissert R. (2015). Peripheral blood mononuclear cells: isolation, freezing, thawing, and culture. Methods Mol. Biol..

[21] Zhu Y., Ning Z., Deng Y., Huang C., Li H., Sun Y. (2026). Enhanced peripheral blood mononuclear cell lipid metabolism drives poor immunotherapy response via ferroptotic stress-induced T cell exhaustion in hepatocellular carcinoma. Interdisc. Med..

[22] Eldalal O., Tabana Y., Babu D., Tran N.H., Lockhart S., Kranrod J. (2026 May 1). Unraveling dimethylformamide-induced neutrophilic differentiation in HL-60 cells: a proteomic and functional comparison with dimethyl sulfoxide. PLoS One.

[23] Bouarroudj H., Gillot C., Decarpentrie J., Mullier F., Douxfils J. (2026). Flow cytometry-based characterization of isolated human neutrophils: evaluating viability, purity and defining a reference interval for basal activation in functional assays. J. Immunol. Methods.

[24] Babu D., Le T.H., Liu H., Raji D., Tran N.H., Lockhart S. (2026). Edaravone as an antioxidant adjuvant to attenuate clozapine toxicity in vitro. Toxicol. Lett..

[25] Krémer V., Godon O., Bruhns P., Jönsson F., de Chaisemartin L. (2023 Nov 24). Isolation methods determine human neutrophil responses after stimulation. Front. Immunol..

