# Supplementary material for: Standardized human granulocyte isolation using Mono-Poly™ resolving medium: protocol development, validation, and comparison with Lymphoprep™
Source: MethodsX. 2026 Oct 16;17:104213. doi: 10.1016/j.mex.2026.104213 (PMC13738167; doi:10.1016/j.mex.2026.104213)
Supplement: Supplementary file 1 [file mmc1.docx]

Supplementary Material

**Evaluating Purity of Neutrophil Isolation: A Comparative Study of Two Density Gradient Methods**

Othman Eldalal ^1,2^, Steven Lockhart1, Huan Liu^1^, John-Paul R Cooper^1^, Dinesh Babu^3^, Arno G. Siraki ^1^ *

^1^ Faculty of Pharmacy & Pharmaceutical Sciences, College of Health Sciences, University of Alberta, Edmonton, Canada.

^2^ Department of Drug Technology, Faculty of Medical Technology, Derna, Libya.

^3^ Li Ka Shing Institute of Virology, University of Alberta, Edmonton, Canada.

Table S1. Search Parameters for Neutrophil Isolation Methods in PubMed and Scopus.

| **Isolation Method** | **Database** | **Search Query String** |
| --- | --- | --- |
| **A: Density Gradient** | PubMed | ((Neutrophil OR "Polymorphonuclear Cell") AND (Isolation OR Purification OR Separation) AND ("Density Gradient" OR Ficoll OR Lymphoprep OR "Mono-Poly" OR Percoll)) AND (2000:2025[DP]) NOT Review[PT] |
|  | Scopus | TITLE-ABS-KEY((neutrophil OR "polymorphonuclear cell") AND (isolation OR purification OR separation) AND ("density gradient" OR ficoll OR lymphoprep OR "mono-poly" OR percoll)) AND PUBYEAR > 1999 AND PUBYEAR < 2026 AND NOT DOCTYPE(re) |
| **B: MACS / Magnetic** | PubMed | ((Neutrophil OR "Polymorphonuclear Cell") AND (Isolation OR Purification OR Separation) AND (MACS OR "Magnetic beads" OR "Negative selection" OR "Positive selection")) AND (2000:2025[DP]) NOT Review[PT] |
|  | Scopus | TITLE-ABS-KEY((neutrophil OR "polymorphonuclear cell") AND (isolation OR purification OR separation) AND (macs OR "magnetic beads" OR "negative selection" OR "positive selection")) AND PUBYEAR > 1999 AND PUBYEAR < 2026 AND NOT DOCTYPE(re) |
| **C: Microfluidics** | PubMed | ((Neutrophil OR "Polymorphonuclear Cell") AND (Isolation OR Purification OR Separation) AND (Microfluidic OR "Chip" OR "Microchannel")) AND (2000:2025[DP]) NOT Review[PT] |
|  | Scopus | TITLE-ABS-KEY((neutrophil OR "polymorphonuclear cell") AND (isolation OR purification OR separation) AND (microfluidic OR chip OR microchannel)) AND PUBYEAR > 1999 AND PUBYEAR < 2026 AND NOT DOCTYPE(re) |

Note: Reviews and abstracts were not included via manual review during deduplication.
